# Study of the effects of cognitive behavioral therapy versus dialectical behavior therapy on executive function and reduction of symptoms in generalized anxiety disorder

**DOI:** 10.47626/2237-6089-2020-0156

**Published:** 2022-07-14

**Authors:** Behrooz Afshari, Fatemeh Jafarian Dehkordi, Ali Asghar Asgharnejad Farid, Behnoosh Aramfar, Zohreh Balagabri, Mohsen Mohebi, Nayereh Mardi, Parisa Amiri

**Affiliations:** 1 Department of Clinical Psychology Kashan University of Medical Sciences Kashan Iran Department of Clinical Psychology, Kashan University of Medical Sciences, Kashan, Iran.; 2 School of Behavioral Sciences and Mental Health Tehran Institute of Psychiatriy Iran University of Medical Sciences Tehran Iran Department of Clinical Psychology, School of Behavioral Sciences and Mental Health, Tehran Institute of Psychiatriy, Iran University of Medical Sciences, Tehran, Iran.; 3 Islamic Azad University Kermanshah Iran Islamic Azad University - Kermanshah Branch, Kermanshah, Iran.; 4 Farhangian University of Kermanshah Kermanshah Iran Farhangian University of Kermanshah, Kermanshah, Iran.

**Keywords:** Generalized anxiety disorder, dialectical behavior therapy, cognitive behavioral therapy, executive function

## Abstract

**Introduction:**

The effects of dialectical behavior therapy on generalized anxiety disorder have not been examined to date, whereas cognitive behavioral therapy is a well-known psychotherapy for generalized anxiety disorder.

**Objectives:**

This study investigated the effects of cognitive behavioral therapy versus dialectical behavior therapy on executive function and reduction of symptoms in generalized anxiety disorder.

**Method:**

In the present study, 72 generalized anxiety disorder patients were randomly assigned to one of two groups: dialectical behavior therapy or cognitive behavioral therapy. Evaluations were performed at baseline, post-test, and three months after interventions as a follow-up. Measures included the Structured Clinical Interview for DSM-IV Axis I disorders, the Generalized Anxiety Disorder scale, the Beck Anxiety Inventory, the Beck Depression Inventory, the Tower of London Task, and the Wisconsin Card Sorting Task.Results: The results of the present study showed that both groups had reduced scores for depression and anxiety and increased scores for executive function after the psychotherapies. These changes were maintained at follow-up.

**Conclusion:**

Although depression and anxiety symptoms were significantly reduced by cognitive behavioral therapy, dialectical behavior therapy was more effective for improving executive function.

## Introduction

Generalized anxiety disorder (GAD) is characterized by significant worry about several events over the past six months. The disorder is difficult to control and is associated with physical symptoms such as restlessness, sleep problems, and muscle tension.^1^ There is a two-to-one ratio of women to men in this disorder. Its comorbid disorders are social phobias, specific phobias, depression, and panic disorder.^2^

Problems with executive function (EF) affecting planning, problem solving, and psychological flexibility have already been confirmed in patients with GAD by numerous studies,^3,4^ but it is not yet entirely clear whether psychotherapy can improve these problems in GAD patients. Executive function is a term that encompasses a set of neuropsychological strategies such as thoughts, psychological flexibility, problem-solving, planning, and emotion regulation, and is also associated with job performance.^5^ Problems with EF may lead to real-life problems that manifest as behavioral problems. Some deficiencies related to EF are related to the symptoms of GAD and helping to correct them will help to reduce the severity of the symptoms of this disorder.^6^

In recent years, many researchers have tried to offer treatments for anxiety disorders, especially GAD. Many studies have shown that although the basic treatment for GAD is medication, about two-thirds of patients continue to show symptoms even after a course of medication. Furthermore, medication has been unable to reduce the severity of anxiety.^7^

Cognitive-behavioral therapy (CBT) aims to reduce the severity of mental disorders by changing behaviors and cognitions.^8,9^ CBT for anxiety disorders mainly focuses on changing thoughts and beliefs using behavioral and cognitive techniques.^10^ A review study reported that CBT improved the symptoms of patients with GAD.^11^ However, while CBT has been effective in reducing negative thoughts in GAD, it has had less effect on GAD than on other anxiety disorders.^12^ In addition, in GAD, worries generally focus on a wide range of issues. The main cause of anxiety is therefore unknown in these patients. Moreover, CBT involves a set of techniques, and it is not clear which technique has the greatest effect on anxiety.^13^

Dialectical behavioral therapy (DBT) is one of the new psychotherapies, focusing on emotion regulation, mindfulness, interpersonal relationships, and distress tolerance, and was initially used to treat borderline personality disorder in which patients suffer from suicidal thoughts and behaviors.^14^ DBT primarily focuses on educating patients to reduce the severity of their emotional problems.^15^ DBT skills and strategies have not yet been used to attempt to improve EF in patients with GAD, but research suggests that these skills may be effective in disorders associated with emotion regulation problems, especially GAD.^16^ To date, DBT has been used for other mental disorders, such as drug-related disorders,^17^ eating disorders, schizophrenia,^18^ bipolar disorder, and generalized anxiety disorder.^19^ However, the effects of DBT for addressing EF problems in patients with GAD have not yet been investigated. Patients with GAD have characteristics such as limited perception of emotional experiences, severe emotional reactions, and inability to apply appropriate coping strategies.^20^ Also, many investigations have shown that DBT can be effective for treating emotional problems as well as disorders characterized by emotion dysregulation.^21^

Emotion regulation means the ability to control emotions. Dysregulation of emotion and related problems are among the issues that DBT deals with. However, one of the differences between DBT and emotion regulation therapy is that, in addition to emotion regulation, DBT also addresses distress tolerance, mindfulness, and interpersonal relationships, which can help patients significantly.^22^ The results of a study of the effectiveness of emotion regulation therapy for GAD showed that this treatment resulted in significant improvements in anxiety, attention inflexibility, quality of life, and emotional problems.^23,24^ Therefore, the present study aimed to compare the effects of DBT and CBT on EF in GAD for the first time.

## Materials and methods

### Participants

**Figure 1:**
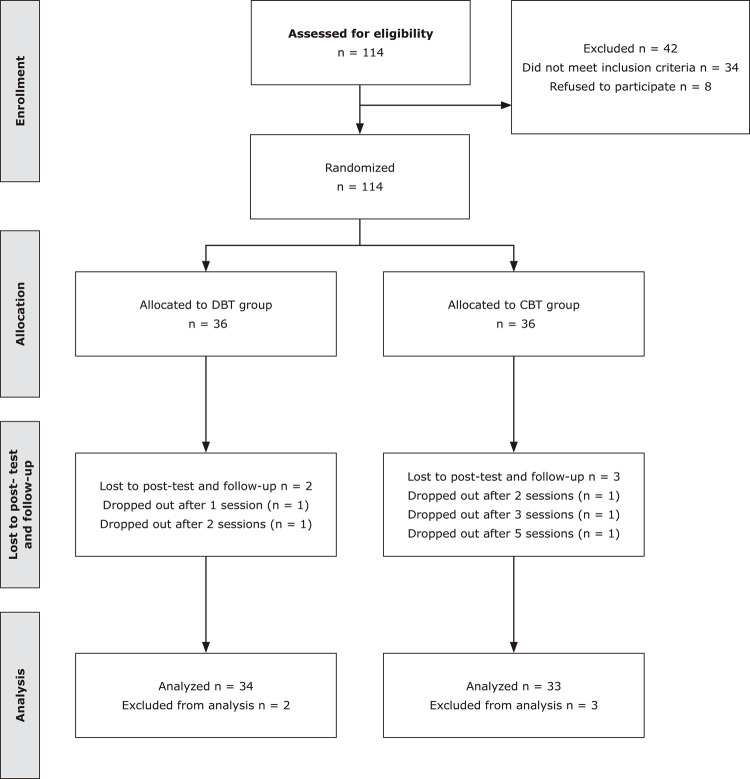
Participant selection flowchart

The study was registered on Iranian Registry of Clinical Trials (IRCT2017031233023N1) and assessed by the Kashan University of Medical Science. Furthermore, informed consent was obtained from all patients. The present study was a randomized control trial in which seventy-two patients with GAD who were referred to a psychiatric hospital located in Iran (Kargarnejad Hospital) from February 2019 to February 2020 were randomly enrolled into one of two groups: the CBT group or the DBT group. All participants had the same probability of being selected for either of the groups. Participants who were allocated to the two groups completed 16 sessions. An expert psychiatrist diagnosed all patients. Inclusion criteria were the presence of GAD, age 18-45 years, having had no prior psychotherapy for at least 6 months before this study, and level of education higher than the eighth grade. Also, exclusion criteria were other major mental disorders (major depression, psychosis, or alcohol or drug use disorder), serious neurological disorders or severe physical illness, and being absent from more than two sessions. The therapy process is illustrated in Figure 1.

### Interventions

#### Cognitive behavioral therapy

This protocol was adapted from Covin et al.^25^ The 16, one-hour sessions focused on relaxation strategies, psychoeducation, behavioral therapy skills, and cognitive strategies. Sessions 1-4 covered identifying GAD-related problems, defining GAD, and introducing the therapy. Sessions 5-6 covered mindfulness. Sessions 7-10 covered cognitive strategies. Finally, sessions 11-16 covered behavioral therapy strategies and integrated techniques from strategies covered previously. The treatment was in an individual format.

#### Dialectical behavioral therapy

This protocol was adapted from the standard DBT manual.^26^ Treatment was delivered in an individual format. Participants in the DBT group received 16, one-hour sessions of DBT. All sessions focused on mindfulness skills to improve management of crises and self-control, distress tolerance, emotion regulation, and interpersonal effectiveness skills. The standard treatment focuses on emotion regulation strategies, consisting of information about emotions and techniques to cope with emotional dysregulation. There were also sessions covering information on interpersonal effectiveness techniques, tolerating painful emotions, and techniques to help patients develop healthy relationships.

## Therapists

The therapists were two professional psychologists; both had been trained in CBT and DBT. The therapists were also supervised by two professional psychologists during the therapy.

## Measures

### Demographic information

Demographics such as age, gender, number of hospitalizations, years of education, age at diagnosis, psychotherapy history, age at first hospitalization, and comorbidity with other disorders were obtained using a demographic questionnaire.

### Structured Clinical Interview for DSM-IV Axis I disorders (SCID-I)

This scale is one of the most famous structured interviews for diagnosing Axis I Disorders. The interview consists of 24 items assessing Axis I disorders.^27,28^ The SCID-I has shown appropriate psychometric characteristics in the Persian population. Furthermore, overall kappa was reported as 0.55 for lifetime diagnosis and 0.52 for current diagnosis.^29^

### Generalized Anxiety Disorder scale (GAD-7)

The GAD-7 was created by Löwe et al. and consists of 7 questions for assessment of GAD. Scores on the scale range from 0-21. The test-retest coefficient of this questionnaire has been reported as 0.83 and its Cronbach’s alpha as 0.92,^30^ while a Cronbach’s alpha coefficient of 0.85 was reported for a Persian community.^31^

### Beck Anxiety Inventory (BAI)

The inventory is a 21-item scale for assessment of anxiety. Scores range from 0-63, where 8-15= mild anxiety, 16-25 = medium anxiety, and 26-63 = severe anxiety.^32,33^ Cronbach’s alpha was 0.88 in a Persian community.^34^

### Beck Depression Inventory (BDI-II)

The inventory is a 21-item scale for assessment of depression. Scores on the scale range from 0-63, where 10-19 = mild depression, 20-28 = medium depression, and 29-63 = severe depression.^35^ Cronbach’s alphas for all subscales ranged from 0.58 to 0.79 in a Persian population.^36^

### Tower of London Task (TOL)

The TOL is a planning task originally designed by Tim Shallice. This task employs three balls of differing colors and scores range from 0 to 36.^37,38^ The TOL is a well-known tool for evaluation of frontal lobe functions and planning abilities in clinical individuals.^39^

### Wisconsin Card Sorting Task (WCST)

This task includes four types of cards that differ by quantity, color, and shape. The WCST assesses cognitive flexibility and problem-solving.^40^ The WCST includes 128 cards. The WCST indicators are correct responses, perseverative errors, and non-perseverative errors. The number of correct responses is related to better function.^41^

## Statistical analyses

The Statistical Package for the Social Sciences (SPSS) version 20 was used to analyze the data. Nominal variables including comorbidity with other disorders and gender were compared using the chi-square test. Analysis of variance (ANOVA) was used to compare means for age, education, GAD-7, BAI, and BDI-II. Analysis of repeated measures was used to compare the CBT and DBT groups at baseline, after the interventions, and after three months of follow-up.

## Results

### Descriptive statistics

The participants were 26 men and 46 women and all participants were ethnic Persians. Participants were randomly allocated to a DBT or a CBT group, including 24 women (66.6%) and 22 women (64.7%) respectively. Additionally, 33 members of the CBT group and 34 members of the DBT group completed the study. Major comorbid disorders included: substance use disorder (8.1%), major depressive disorder (16.2%), and panic disorder (17.5%). Thirty-one of the 72 patients with GAD had previously engaged in psychotherapy. All demographic information is provided in Table 1.

**Table 1:** Demographic features of the sample

Characteristics	DBT group (n = 36)	CBT group (n = 36)	P-value
Age, mean (SD)	27.12 (5.65)	27. 44 (5.24)	12.2^*F*^
Female patients	66.6% (n = 24)	64.7% (n = 22)	1.4^*χ^2^*^
Marital status (single vs. married vs. divorced or separated)	n = 17/17/2	n = 16/17/3	1.2^*χ^2^*^
Years of education, mean (SD)	11.04 (3.06)	10.74 (3.02)	21.4^*F*^
Age at diagnosis, mean (SD)	21.46 (6.18)	20.72 (5.10)	2.3^t^
Number with comorbid diagnoses (%)	72.2% (n = 26)	66.6% (n = 24)	1.3^*χ^2^*^
Number with previous engagement with psychotherapy (%)	47.2% (n = 17)	38.8% (n = 14)	1.1^*χ^2^*^

CBT = cognitive behavioral therapy; DBT = dialectical behavior therapy; SD = standard deviation.

### Anxiety symptoms

Anxiety ranges reduced over time in both the DBT and the CBT groups three months post-intervention and nearly maintained the reduction at three months’ follow-up. In general, BAI and GAD-7 scores diminished over time in both groups, demonstrating decreased anxiety severity after interventions. Although participants had symptom levels above the cut-offs at baseline, after the interventions they had symptom severity below the cut-offs. Furthermore, the CBT group exhibited greater change than the DBT group (Tables 2 and 3).

**Table 2:** Descriptive statistics for the measures over the three-time periods assessed in this study, by condition

Measure	DBT group (n = 34) Mean (SD) [change from pre-treatment as *d*]			CBT group (n = 33) Mean (SD) [change from pre-treatment as *d*]		
	Pre-treatment	Post-treatment	Follow-up	Pre-treatment	Post-treatment	Follow-up
GAD-7	19.46 (6.28)	12.22 (3.42) [0.92]	13.14 (4.33) [0.78]	16.41 (6.42)	7.43 (2.45) [0.84]	7.23 (2.50) [0.82]
BAI	31.27 (7.24)	15.89 (3.28) [0.95]	14.55 (4.36) [0.89]	32.31 (6.47)	7.94 (6.24) [0.77]	12.36 (6.27) [0.81]
BDI-II	14.62 (5.39)	8.46 (3.86) [1.07]	8.63 (4.74) [0.87]	13.46 (5.21)	6.11 (4.37) [0.76]	7.64 (4.52) [0.73]
TOL	18.00 (3.48)	29.19 (5.42) [0.85]	28.23 (3.36) [0.84]	17.92 (3.45)	24.03 (3.57) [1.23]	21.62 (3.28) [1.04]
WCST						
Trials correct	68.57 (10.42)	86.30 (13.21) [0.87]	79.85 (15.74) [0.78]	65.83 (10.15)	71.86 (10.16) [0.74]	69.66 (10.23) [0.92]
Trial errors	59.43 (10.21)	42.69 (16.28) [0.75]	48.15 (11.16) [0.77]	62.17 (10.26)	56.14 (10.43) [0.72]	58.24 (11.64) [0.83]
Perseverative	27.88 (14.54)	14.31 (14.14) [0.74]	18.61 (9.25) [0.92]	38.68 (12.37)	23.62 (8.36) [0.79]	23.45 (7.26) [0.76]
Non-perseverative	31.12 (10.16)	27.69 (10.18) [0.82]	29.49 (10.13) [0.81]	23.32 (11.41)	32.38 (9.65) [0.65]	34.55 (12.46) [0.82]

BAI = Beck Anxiety Inventory; BDI-II = Beck Depression Inventory-II; CBT = cognitive behavioral therapy; DBT = dialectical behavior therapy; GAD-7 = Generalized Anxiety Disorder scale; SD = standard deviation; TOL = Tower of London computerized task; WCST = Wisconsin Card Sorting Task.

**Table 3:** Main effects and interactions from the mixed-effects ANOVA computed in this study

Measure	Main effect of time	Main effect of condition	Condition-time interaction
GAD-7	*F*(2, 144) = 13.08, p < .001, *f*^2^ = 0.50	*F*(1, 72) = 1.72, p = .146, *f*^2^ = 0.16	*F*(2,144) = 12.32, p < .001, *f*^2^ = 0.37
BAI	*F*(2, 144) = 20.11, p < .001, *f*^2^ = 0.50	*F*(1, 72) = 1.78, p = .184, *f*^2^ = 0.16	*F*(2,144) = 14.36, p < .001, *f*^2^ = 0.43
BDI-II	*F*(2, 144) = 28.25, p < .001, *f*^2^ = 0.63	*F*(1, 72) = 6.48, p = .011, *f*^2^ = 0.31	*F*(2,144) = 21.54, p < .001, *f*^2^ = 0.54
TOL	*F*(2, 144) = 23.64, p < .001, *f*^2^ = 0.20	*F*(1, 72) = 20.54, p < .001, *f*^2^ = 0.53	*F*(2,144) = 13.91, p < .001, *f*^2^ = 0.44
WCST			
Trials correct*	*F*(2, 144) = 14.03, p < .001, *f*^2^ = 0.54	*F*(1, 72) = 27.08, p < .001, *f*^2^ = 0.59	*F*(2,144) = 13.75, p < .001, *f*^2^ = 0.54
Trial errors*	*F*(2, 144) = 14.03, p < .001, *f*^2^ = 0.54	*F*(1, 72) = 27.08, p < .001, *f*^2^ = 0.59	*F*(2,144) = 13.75, p < .001, *f*^2^ = 0.54
Perseverative	*F*(2, 144) = 7.32, p = .002, *f*^2^ = 0.35	*F*(1, 72) = 9.15, p = .004, *f*^2^ = 0.38	*F*(2,144) = 7.34, p = .002, *f*^2^ = 0.35
Non-perseverative	*F*(2, 144) = 4.68, p = .004, *f*^2^ = 0.27	*F*(1, 72) = 0.87, p = .575, *f*^2^ = 0.17	*F*(2,144) = 4.60, p = .004, *f*^2^ = 0.27

BAI = Beck Anxiety Inventory; BDI-II = Beck Depression Inventory-II; GAD-7 = Generalized Anxiety Disorder scale; SD = standard deviation; TOL = Tower of London computerized task; WCST = Wisconsin Card Sorting Task.

* Differences are the same for WCST trials correct and in error because of the interdependence of the variables.

### Depression symptoms

Depression ranges reduced over time in both the DBT and the CBT groups three months post-intervention and nearly maintained the reduction at three months’ follow-up. Furthermore, the CBT group exhibited greater change than the DBT group (Tables 2 and 3).

### Problem-solving and cognitive flexibility

All subscales of the WCST, including average error, non-perseverative error, perseverative error, and correct responses changed over time in both the DBT and the CBT groups, demonstrating improved cognitive flexibility and problem-solving, and these changes remained nearly the same after three months of follow-up. Changes were greater in the DBT group than in the CBT group for all WCST subscales.

### Planning and goal-directed behaviors

There was improvement across EF measures in both the DBT and the CBT groups. Higher scores indicated better planning after three months of intervention and the improvement was nearly maintained at three months of follow-up. All subscales had varied more in the DBT group than in the CBT group after the interventions.

## Discussion

Psychological psychotherapies such as DBT and CBT are effective and efficient for solving psychological problems. This is the first study to compare the effects of DBT and CBT on EF and symptom severity in GAD. In this study, we aimed to evaluate the effectiveness of DBT and CBT for alleviating anxiety and depression symptoms and improving goal-directed behaviors, planning, problem-solving, and psychological flexibility. The results of this study showed that DBT led to improvement of all research variables in patients with GAD in the DBT group.

The results of the present study showed that DBT has a greater effect on the EF of patients with GAD than CBT. DBT primarily focuses on emotion regulation. Problems with emotion regulation are among the main problems in GAD and can lead to other problems. If patients can improve their emotion regulation, then problems associated with the severity of their symptoms are more likely to go away. DBT can improve concentration and awareness by regulating emotion.^42^ In this case, improved focus helps to improve EF. Emotion regulation plays a key role as a mediating variable. Additionally, the results of this study showed improved EF among members of the DBT group. These functions have significant effects on behavior, life satisfaction, relationships, and job performance.^5^ Thus, through its emotion regulation techniques, DBT can improve EFs such as problem-solving and planning as well as job performance and life satisfaction in patients with GAD.

The ability to regulate emotion can also be enhanced step by step throughout life. We can also teach skills to adults to help improve their quality of life. Therefore, emotion regulation strategies are important for reducing inappropriate behaviors and promoting appropriate behaviors.^43^ Renna et al. reported that emotion regulation therapy improved anxiety symptoms, social disability, quality of life, attentional flexibility, and reappraisal in 31 GAD patients.^24^ Hence, emotion regulation is considered one of the more effective DBT techniques.

This study, like any other study, has its limitations. One of these limitations is the small sample size. It is suggested that a larger sample size be used in future studies to enable generalization of the findings. Furthermore, this study was conducted in Iran and the subject should be studied in other communities. Finally, it would be good to use DBT for other anxiety disorders such as specific phobias and social phobia and compare its effects with those of CBT and third-wave behavioral therapies.

## Conclusion

In this study, the effects on GAD of DBT and CBT were compared. The results showed that although CBT reduced symptoms of anxiety and depression, DBT led to improved EF in patients with GAD. These findings will be useful to support future research into use of psychotherapy for GAD. Also, the application of DBT to other mental disorders that involve problems with emotion regulation and EF should be able to improve therapy for mental disorders and reduce the severity of mental disorders.
